# Effect of BMI on Operative Time in Patients Undergoing Distal Pancreatectomy

**DOI:** 10.7759/cureus.88545

**Published:** 2025-07-22

**Authors:** Jesse P Caron, Michelle Horowitz, William J Cobb, Jianbin Zhu, Armando Rosales

**Affiliations:** 1 General Surgery, AdventHealth Orlando, Orlando, USA; 2 Biostatistics, AdventHealth Orlando, Orlando, USA; 3 Surgical Oncology, AdventHealth Orlando, Orlando, USA

**Keywords:** body mass index, distal pancreatectomy, obesity, operative time, preoperative planning

## Abstract

Background

Preoperative body mass index (BMI) is known to impact surgical outcomes, but its effect on operative time in distal pancreatectomy (DP) remains unclear. This study hypothesizes that higher BMI is associated with prolonged operative time in DP, regardless of surgical approach.

Methods

A retrospective review of 48 patients who underwent DP, with or without splenectomy, at Advent Health Orlando in Orlando, Florida (October 2019-April 2024) was conducted. Patients were categorized by surgical approach: laparoscopic, robotic, robotic conversion to open (planned or unplanned), and open. Laparoscopic hand-assisted cases were excluded. Data included preoperative (age, sex, BMI, and American Society of Anesthesiologists (ASA) class) and intraoperative variables (operative time, splenectomy status, blood loss, conversion to open, and ICU admission). Pearson correlation and linear regression assessed BMI’s impact on operative time.

Results

The mean BMI was 28.9 (range: 19.8-44.7), with 31.3% classified as obese (BMI ≥ 30). BMI correlated with longer operative time (r = 0.333, p = 0.021), although BMI categories (normal <25, overweight 25-30, obese >30) showed no significant difference (p = 0.24). Furthermore, this correlation persisted after controlling for surgical approach, tumor size, prior abdominal surgery, tumor location, ASA class, and concurrent splenectomy. BMI was not associated with conversion to open surgery, blood loss, or splenectomy rates. ICU admission was significantly associated with BMI when analyzed continuously (yes: 44.9 ± 7.6; no: 28.1 ± 5.5; p = 0.008), but not categorically (p = 0.22).

Conclusion

Higher BMI is significantly associated with increased operative time in DP, underscoring the need for preoperative planning in patients with elevated BMI. BMI as a continuous variable provides greater predictive value for surgical outcomes than categorical classification.

## Introduction

Obesity has been linked to adverse surgical outcomes, including increased rates of postoperative infection, venous thromboembolism, and renal complications [1]. These findings have driven the adoption of preoperative strategies aimed at mitigating the risks associated with obesity, including caloric restriction and weight optimization programs [2]. However, the urgent nature of surgical oncology oftentimes does not afford the time necessary to maximally optimize patients prior to surgery.

Current research indicates that obesity does impact pancreatic surgery outcomes, but the extent and nature of this effect remain controversial. While some studies report increased intraoperative complexity and complications in obese patients, others suggest no significant differences in short-term outcomes. Interestingly, the reported improved long-term survival in obese patients compared to normal-weight individuals has been coined the "obesity paradox," which refers to the phenomenon where, despite obesity being an established risk factor for certain cancers, several studies have suggested that obese patients (body mass index (BMI) >30) have better cancer-specific outcomes than non-obese patients. However, this paradox remains controversial due to potential confounding factors such as differences in tumor biology, lead-time bias from increased medical surveillance in obese patients, and limitations in using BMI as a measure of adiposity, as it does not account for body composition differences like sarcopenic obesity, which may influence outcomes [3].

These discrepancies highlight the complexity of assessing obesity’s impact on surgical outcomes, as patient selection, perioperative care, and institutional differences may also contribute to variations in findings. While obesity is clearly associated with altered perioperative risks, its overall impact on morbidity and mortality remains debated due to these methodological and biological uncertainties.

While the effects of obesity on operative time and postoperative outcomes of pancreaticoduodenectomy (PD) for pancreatic cancer have been studied, the impact of BMI on operative time in distal pancreatectomy (DP), with or without splenectomy, for any indication, remains understudied. Prolonged operative time is particularly relevant in DP, given the procedure’s technical complexity and variability in surgical approach.

The primary aim of this study is to investigate whether increased BMI correlates with prolonged operative time in DP, irrespective of surgical approach. Specifically, we hypothesize that BMI is positively associated with operative time, highlighting the need for tailored preoperative planning in this population. Secondary objectives include conversion to open surgery, intraoperative blood loss, splenectomy rates, admission or readmission rates, infection rates, ICU admission, and mortality rates.

## Materials and methods

Materials and methods

This study was conducted in adherence to the STROBE (Strengthening the Reporting of Observational Studies in Epidemiology) guidelines for observational studies. The study was a retrospective cohort analysis of 48 patients who underwent DP, with or without splenectomy, at AdventHealth Orlando in Orlando, Florida, between October 2019 and April 2024. Institutional Review Board (IRB) approval was obtained prior to data collection (approval no. 1353509), and the study adhered to ethical principles as outlined in the Declaration of Helsinki. Given the retrospective nature of this study, the IRB waived the requirement for individual patient consent. All patient data were handled with strict confidentiality in accordance with the Health Insurance Portability and Accountability Act (HIPAA) guidelines and institutional policies.

Study population

**Figure 1:**
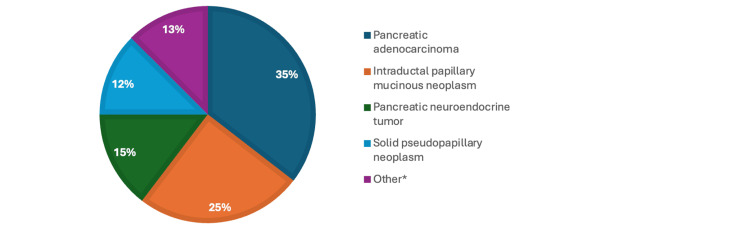
Surgical Indications for distal pancreatectomy with or without splenectomy. Figure 1 illustrates the surgical indications for distal pancreatectomy with or without splenectomy. The most common indication in this dataset is pancreatic adenocarcinoma (n = 17), followed by intraductal papillary mucinous neoplasm (n = 12) and pancreatic neuroendocrine tumor (n = 7). Solid pseudopapillary neoplasm (n = 6) and other conditions (n = 6) also contributed to the need for surgery. The "other" category includes lymphoepithelial cyst, mucinous cystic neoplasm, and metastatic cancer. This distribution highlights the diverse pathology that necessitates pancreatic resection.

The inclusion criteria encompassed all adult patients undergoing DP for any indication between October 2019 and April 2024 by one of two surgeons performing DP. Patients were excluded if relevant data, such as preoperative BMI or operative time, were incomplete, or if the patient did not undergo a DP. From October 2019 to April 2023, the surgical approach was laparoscopic DP, and from May 2023 to April 2024, it was robotic-assisted. In both minimally invasive approaches, the clockwise technique was used, and the pancreas was divided using the squeeze technique with an Echelon 60 linear stapler with reinforcement, depending on pancreas thickness; either a blue or green load was utilized accordingly. Surgical indications included benign and malignant pancreatic pathologies. The breakdown of indications for distal pancreatectomy, with or without splenectomy, is provided in Figure 1.

Data collection

Data were extracted from the institution’s electronic medical records. Preoperative variables included age, sex, ethnicity, prior abdominal surgery, ASA class, and BMI. Intraoperative data collected included surgical approach (laparoscopic, robotic, robotic planned conversion to open, robotic unplanned conversion to open, and open techniques), operative time, splenectomy status, tumor size and location, intraoperative blood loss, rate of conversion to open, and postoperative ICU admission. BMI was analyzed as both a continuous and categorical variable (normal <25 kg/m², overweight 25-30 kg/m², and obese >30 kg/m²). The data were collected from a chart review by the research team, and statistical analysis expertise was available to the authors who performed the statistical analyses.

Statistical analysis

Statistical analyses were conducted by an institutional biostatistician. A simple linear regression with Pearson correlation coefficient was used to identify the effects of BMI on two outcomes of operative time and intraoperative blood loss, respectively. The Shapiro-Wilk test was used to test the normality of two outcomes and BMI. No normality assumption was satisfied. Thus, a nonparametric test was used to compare BMI between or across open procedures, admission/readmission, and mortality, using the Mann-Whitney test (two groups) and the Kruskal-Wallis test (three or more groups). In addition, Chi-square or Fisher's exact test was used to find the association between these variables with the three BMI groups. A p-value <0.05 was considered statistically significant. IBM SPSS Statistics for Windows, version 29.0 (IBM Corp., Armonk, NY) was used for all analyses.

## Results

A total of 48 patients who underwent DP, with or without splenectomy, were included in the analysis. Table 1 shows descriptive statistics including mean and standard deviation (SD) values of age, BMI, and operative time. In this analysis of 48 patients, the mean age was 61.8 years, the mean BMI was 28.9 kg/m² (SD = 6.0, range: 18.7 to 44.4), and the mean operative time was 211.7 minutes with a SD of 82.7.

**Table 1:** Descriptive statistics

	N	Minimum	Maximum	Mean	Std. deviation
Age at surgery (years)	48	25.0	82.0	61.8	15.4
BMI (kg/m^2^)	48	18.7	44.4	28.9	6.0
Operative time (minutes)	48	90	440	211.7	82.7

**Figure 2:**
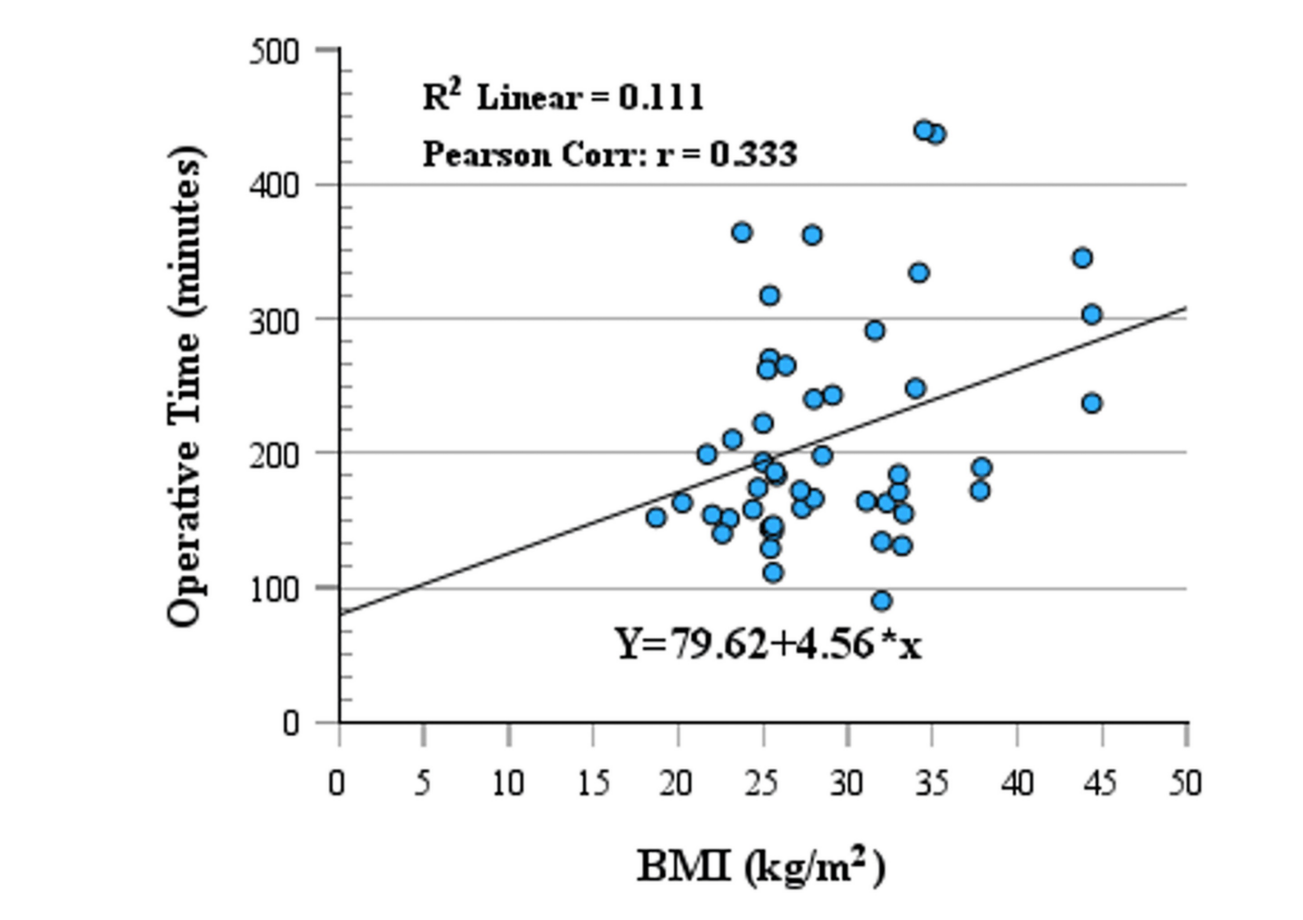
BMI vs. operative time in distal pancreatectomy with or without splenectomy Figure 2 demonstrates the positive linear relationship between BMI (kg/m^2^) and operative time (minutes) in patients undergoing distal pancreatectomy (DP). The Pearson correlation coefficient (r = 0.333, p = 0.021) indicates a statistically significant moderate correlation, suggesting that a higher BMI is associated with longer operative time. This relationship is represented by the following equation: operative time = 79.62 + 4.56 x BMI.

**Figure 3:**
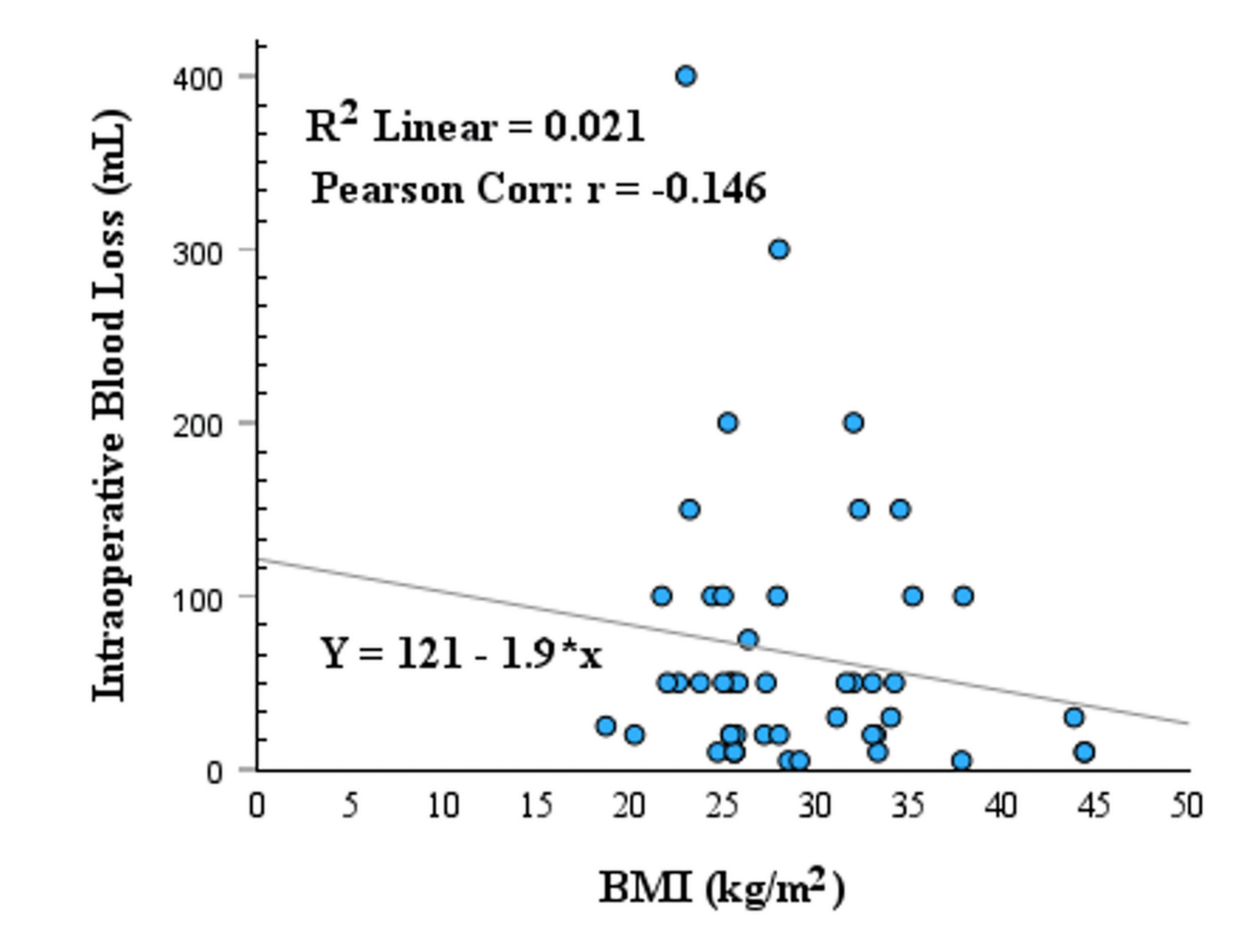
BMI vs. intraoperative blood loss in distal pancreatectomy with or without splenectomy Figure 3 demonstrates the negative linear relationship between BMI (kg/m^2^) and intraoperative blood loss (mL) in patients undergoing distal pancreatectomy (DP). The Pearson correlation coefficient (r = -0.146, p = 0.32) indicates a non-statistically significant moderate correlation, suggesting that BMI has no linear relationship with intraoperative blood loss. This relationship is represented by the equation: intraoperative blood loss = 121 – 1.9 x BMI.

The Pearson correlation coefficients for BMI and operative time and BMI and intraoperative blood loss were shown by the scatter plot with a linear regression line in Figure 2 and Figure 3, respectively. The Pearson correlation coefficients for BMI and operative time were 0.333, indicating a significant positive linear relationship between BMI and operative time (p = 0.021). The Pearson correlation coefficients for BMI and intraoperative blood loss were -0.146, indicating a non-significant negative linear relationship between BMI and intraoperative blood loss (p = 0.32). The Pearson correlation coefficients are also summarized in Table 2 with significance levels. The comparisons of operative time and intraoperative blood loss across BMI groups (normal: BMI <25, n = 10 (20.8%); overweight: BMI 25-30, n = 20 (41.7%); obesity: BMI >30, n = 18 (37.5%)), with significance levels determined by the Kruskal-Wallis test, are shown in Table 2. The relationship between BMI and categorical outcomes, including conversion to open procedures, wound infection, and postoperative complications, is summarized in Table 3.

**Table 2:** Relationship between BMI and continuous outcomes. Table 2 shows the relationships between BMI and outcomes with operative time and intraoperative blood loss. The overall mean value presents the mean ± standard deviation of the two variables. The correlation coefficient and p display the correlations between BMI and these two variables with significance levels by Student’s T-test. The BMI group and p present the mean ± standard deviation for these two variables across BMI groups with significance levels by Kruskal-Wallis test. It shows a significant positive correlation between BMI and operative time with p = 0.021, but no significant association was found when BMI was categorized (p = 0.34), likely due to the small sample size.

Variables	Overall mean value	Correlation coefficient	p	BMI group, n(%)			p
				Normal 10(20.8)	Overweight 20(41.7)	Obesity 18(37.5)	
Operative time (minutes)	211±82	0.333	0.021	187±66	206±66	233±104	0.34
Intraoperative blood loss (mL)	66±78	-0.146	0.32	96±115	58±74	59±57	0.41

Furthermore, we compared BMI between or across open procedures, admission/readmission, and mortality and analyzed the associations between BMI groups and these variables. The results are shown in Table 3. No significant associations were found, indicating that conversion to open procedures, intraoperative blood loss, and splenectomy rates were not significantly affected by BMI.

**Table 3:** Relationship between BMI and categorical outcomes. Table 3 shows the association between BMI/BMI groups and other outcomes.  The overall presents frequencies and percentages. The mean BMI and p display the mean values of BMI by each level and compare the difference with significance level by Mann-Whitney test (two groups) and Kruskal-Wallis test (four groups). The BMI group and p present frequencies and percentages across BMI groups with significance levels by Chi-square test or Fisher's exact test. No significant association was found between BMI and all these outcomes.

Variables	Overall, n(%) 48(100)	Mean BMI	P	BMI group, n(%)			p	Chi-square values
				Normal 10(20.8)	Overweight 20(41.7)	Obesity 18(37.5)		
Conversion to open			0.29				0.79	0.47
Yes	16(33.3)	27.5±4.9		4(40.0)	7(35.0)	5(27.8)		
No	32(66.7)	29.7±6.5		6(60.0)	13(65.0	13(72.2)		
Wound infection (n = 47)			0.86				1.0	0.51
Yes	2(4.2)	28.7±6.2		0(0)	1(5.0)	1(5.6)		
No	45(93.8)	29.0±6.2		9(100)	19(95.0)	17(94.4)		
Reoperation (n = 47)			0.37				1.0	0.28
Yes	3(6.3)	26.8±3.7		1(10.0)	1(5.3)	1(5.6)		
No	44(91.7)	29.1±6.2		9(90.0)	18(94.7)	17(94.4)		
Readmission			0.56				0.85	0.57
Yes	14(29.2)	29.7±5.9		2(20.0)	6(30.0)	6(33.3)		
No	34(70.8)	28.6±6.2		8(80.0)	14(70.0)	12(66.7)		
ICU admission			-				-	-
Yes	-	-		-	-	-		
No	48(100)	28.9±6.0		10(100)	20(100)	18(100)		
Mortality within 30 days			-				-	-
Yes	-	-		-	-	-		
No	48(100)	28.9±6.0		10(100)	20(100)	18(100)		
In-hospital mortality			0.13				0.21	3.88
Yes	1(2.1)	21.7		1(10.0)	0(0)	0(0)		
No	47(97.9)	29.1±6.0		9(90.0)	20(100)	18(100)		
Deep infection			0.27				0.13	4.16
Yes	7(14.6)	31.9±7.2		1(10.0)	1(5.0)	5(27.8)		
No	41(85.4)	28.4±5.8		9(90.0)	19(95.0)	13(72.2)		
Post-operative pancreatic fistula			0.12				0.10	10.52
1	25(52.1)	28.1±6.0		6(60.0)	13(65.0)	6(33.3)		
2	17(35.4)	29.2±6.6		3(30.0)	7(35.0)	7(38.9)		
3	4(8.3)	34.0±1.2		0(0)	0(0)	4(22.2)		
4	2(4.2)	27.8±4.7		1(10.0)	0(0)	1(5.6)		
Exocrine pancreatic insufficiency (n = 47)			1.0				0.51	2.12
Yes	4(8.3)	28.3±4.7		0(0)	3(15.0)	1(5.6)		
No	43(89.6)	29.1±6.2		9(100)	17(85.0)	17(94.4)		

## Discussion

Although obesity has been consistently shown to increase operative time and short-term complications in gastroenterological surgery, it has not consistently been associated with worsened long-term outcomes or mortality [4]; its role as a prognostic factor in pancreatic surgery remains controversial [5]. This study identified a statistically significant, modest correlation between increased BMI and longer operative time in patients undergoing distal pancreatectomy, which persisted after controlling for type of surgical approach, tumor size, prior abdominal surgery, tumor location, ASA class, and presence or absence or concurrent splenectomy. This aligns with findings by Tsai et al. (2010), who reported that obese patients undergoing PD had increased blood loss (p < 0.001) and a higher risk of pancreatic fistula (p = 0.01), although no significant differences in overall complications (p = 0.40) or mortality (p = 0.34) were observed [6]. By contrast, El Nakeeb et al. (2014) observed worse outcomes in overweight patients, including longer operative times (p = 0.003), increased complications (CMJ1) (p = 0.001), higher pancreatic fistula rates (p = 0.0001), delayed gastric emptying (p = 0.008), pulmonary complications (p = 0.04), longer hospital stays (p = 0.0001), and higher mortality (p = 0.001) [7]. Our results suggest that higher BMI may pose technical challenges during pancreatic surgery. The findings align with existing literature indicating that obesity contributes to longer operative times due to factors such as difficulty in visualization, exposure, and access. Given the retrospective design and limited sample size, these associations should be interpreted as hypothesis-generating rather than evidence of causality.

Obesity has been linked to longer operative times, increased blood loss, and increased leak rates in minimally invasive colorectal surgery [8] and has been shown to be an independent risk factor for postoperative complications in ventral hernia repair [9,10]. However, obesity-related complications in pancreatic surgery are still emerging. Ramsey (2011) reported that pooled analyses from a meta-analysis, involving 2,736 patients, demonstrated a significant link between pancreatic fistula and BMI [11]. This finding may be driven by the association between increased BMI and soft pancreatic consistency, a known risk factor for pancreatic fistula [12,13]. In our own cohort, there was no meaningful statistical relationship between BMI and pancreas texture. In addition, increased intraabdominal fat adds technical challenge to the operation, both in terms of abdominal domain and ability to visualize structures.

Our findings of higher BMI having a positive relationship with operative time highlight an important part of the surgical care of overweight and obese patients. A 2018 retrospective cohort study analyzed over 10,000 patients undergoing PD or DP and found that increased operative times were negatively associated with overall morbidity, major complications, pancreatectomy-specific complications, infectious complications, and prolonged hospital stay [14]. Higher operative times also increase risk of venous thromboembolic events, especially for patients undergoing cancer operations [15].

Our study did not show BMI was associated with increased conversion to open, intraoperative blood loss, or fistula rates (Table 2). However, increased BMI did show a significant mean difference in ICU readmission when analyzed as a continuous variable, but not as a categorical variable. The increased ICU readmission rate in patients with higher BMI may be due to the additional comorbidities in patients with metabolic syndrome that contribute to postoperative issues requiring a higher level of care. This finding may suggest that, by considering BMI as a continuous variable, even minor increases in BMI can impact surgical efficiency and postoperative course. While BMI category did not show significant differences in intraoperative outcomes such as blood loss, conversion rates, or the need for splenectomy. While BMI category did not show significant differences in intraoperative outcomes such as blood loss, conversion rates, or the need for splenectomy, these findings, along with the existing literature, support that BMI is an important factor to consider during preoperative planning to anticipate potential complications and optimize surgical approach.

Lastly, our findings highlight the importance of addressing obesity in surgical clinics prior to surgery. Preoperative weight loss in obese patients has been shown to improve 30-day mortality after bariatric surgery, potentially by reducing surgical complexity, improving glycemic control, lowering risk of thrombotic events, and improving respiratory function [16]. For this reason, structured weight loss programs before surgery are of increasing interest. Simple, age-old techniques such as energy restriction are being proposed as cost-effective, timely strategies to reduce weight ahead of elective surgery [17]. However, rapid weight loss with a calorie deficit in a nonmonitored setting may lead to both lean muscle and fat loss. Efforts have been made to identify strategies to target fat loss rather than overall weight loss in order to preserve lean muscle mass [18]. Unlike elective surgery, oncologic operations pose a unique challenge given the urgent need to intervene, thus limiting the ability to fully optimize patients, especially from a weight loss standpoint. A recent meta-analysis published in Annals of Surgical Oncology determined that a prehabilitation program improved outcomes in surgical oncology patients undergoing major abdominal operations [19]. This meta-analysis addressed programs that were aimed at optimizing patients by means of improving functioning capacity through aerobic and anaerobic exercise in addition to nutrition programs that emphasized increased protein intake in an effort to preserve muscle mass. Increasing muscle mass preoperatively has been shown in their population to attenuate postoperative catabolism that contributes to deconditioning postoperatively. Research is needed to investigate strategies to safely and efficiently optimize this cohort of patients undergoing pancreatic surgery specifically.

Limitations

This study is limited by its retrospective design, single-center setting, and relatively small sample size of 48 patients. As a result, the findings may not be generalizable to a broader population. The data were collected from a single institution among only two surgeons, which may contribute to institutional bias, variation in surgical techniques, or differences in postoperative care. Although only two surgeons were involved, differences in technical skill or intraoperative decision-making may still introduce performance bias. Furthermore, while BMI was correlated with operative time when controlling for variables of operative approach, tumor size, prior abdominal surgery, tumor location, ASA class, and presence or absence or concurrent splenectomy, other confounders, such as tumor complexity, surgeon experience, and anesthesia management, were not fully accounted for. Variation in perioperative protocols such as ICU readmission thresholds or intraoperative fluid management may also impact the outcomes observed. Perioperative protocols such as fluid management, thromboembolic prophylaxis, and ICU disposition criteria were not standardized and may have introduced variability not captured in the analysis. Multivariate analysis was not performed, further limiting our ability to adjust for potential confounding. A multi-center, prospective study with a larger cohort would help validate these findings. In addition, this study focused on short-term postoperative outcomes, and future research should assess long-term outcomes, such as complication rates, recurrence, and quality of life in obese patients undergoing pancreatic surgery.

## Conclusions

Higher BMI is associated with longer operative times in distal pancreatectomy with or without splenectomy and a higher likelihood of requiring postoperative ICU care. These findings highlight the importance of considering BMI as a continuous risk factor for surgical planning and postoperative care in pancreatic surgery. However, given the small sample size, single-institution data, and lack of multivariate modeling, these results should be interpreted as exploratory and hypothesis-generating. Future research should explore the impact of obesity on other pancreatic procedures and investigate strategies including oncologic prehabilitation to minimize the effect of BMI on surgical outcomes, including the role of minimally invasive techniques.
